# Classical and Bayesian inference for the new four-parameter Lomax distribution with applications

**DOI:** 10.1016/j.heliyon.2024.e25842

**Published:** 2024-02-05

**Authors:** Mahreen Abdullah, Muhammad Ahsan-ul-Haq, Abdullah M. Alomair, Mohammed A. Alomair

**Affiliations:** a School of Statistics, Minhaj University Lahore, Lahore Pakistan; b College of Statistical Sciences, University of the Punjab, Lahore Pakistan; c Department of Quantitative Methods, School of Business, King Faisal University, 31982, Al-Ahsa, Saudi Arabia

**Keywords:** Generalization, power Lomax, Moments, Metropolis–Hasting's algorithm, Inference, Data analysis

## Abstract

In this study, a new four-parameter Lomax distribution is proposed using a new alpha power transformation technique. The new distribution is named "New Alpha Power Transformed Power Lomax Distribution." Mathematical properties, including moments, the moment-generating function, the mean residual life, order statistics, and the quantile function, are obtained. The maximum likelihood estimation approach is used to estimate the model parameters. A comprehensive simulation is used to evaluate the behavior of maximum likelihood estimators. Two real-world data sets were used to demonstrate the significance of the proposed model, and the results show that the new model performs better when interpreting lifetime data sets. In the end, for the data sets, Bayesian estimation and Metropolis-Hasting's approach were also utilized to construct the approximate Bayes estimates, and convergence diagnostic methods based on Markov Chain Monte Carlo techniques were applied.

## 1 Introduction

The significance of statistical theory stems from its capacity to analyze different types of data sets. Lifetime data modeling is imperative in many arenas, including medicine, engineering, insurance, finance, and many more. It is very common practice to model lifetime data sets using probability distributions and their generalizations. Finding adaptable probability models to accommodate the analysis of varied data types with extreme observations is a difficult task for researchers. However, the traditional probability models do not provide the best fit when the data sets are heavily tailing or contain extreme observations. The addition of one or two parameters to the fundamental distributions encourages the development of new distribution theory modeling ideas. In recent years, extended distributions have been a common method for the derivation of flexible distributions that may be tailored to actual data.

Lomax distribution [1][2][3][4][5]

Several attempts to introduce or generalize the Lomax distribution have been made in recent years, for example, Poisson-Lomax [6], Marshall–Olkin extended Lomax [7], transmuted Lomax [8], extended Lomax [9], gamma-Lomax [10], Weibull Lomax [11], transmuted Weibull Lomax [12], Power Lomax [13], inverse power Lomax [14], weighted Lomax [15], Gumbel-Lomax [16], Marshall–Olkin power Lomax [17], Nadarajah–Haghighi Lomax [18], minimum Lindley Lomax [19], record-based transmuted power Lomax [20], Maxwell–Lomax [21], and discrete Gamma-Lomax distribution [22], etc.

Power Lomax (PLx) distribution was originally introduced by Ref. [13]. The cumulative distribution function (cdf) of the PLx distribution is given by

(1) $$G\left(x;\theta ,\beta ,\lambda \right)=1-\lambda^{\theta }{\left(\lambda +x^{\beta }\right)}^{-\theta },x>0$$

The probability density function (pdf) corresponding to equation (1) is

$$g\left(x;\theta ,\beta ,\lambda \right)=\theta \beta \lambda^{\theta }x^{\beta -1}{\left(\lambda +x^{\beta }\right)}^{-\theta -1}\text{,}$$

On the other hand, there is a substantial body of statistical research on strategies for introducing new families of distributions. Notable among them are the Beta-G family [23], Kumaraswamy-G family [24], Weibull-G family [25], generalized odd log-logistic [26], Alpha power transformation family [27], odd Lindley-G family [28], Odd Fréchet-G family [29], Marshall–Olkin alpha power family [30], generalized odd Burr III-G family [31], Sine Topp-Leone-G family [32], Arcsine-X family [33], Odd Chen-G family [34] and dual-Dagum family [35].

Moreover [36], has introduced the approach of inducing a new parameter to the baseline distribution. The resulting approach is called the New Alpha Power Transformed (NAPT) family, and its pdf and cdf are given by the ensuing formulae,

$$f\left(x\right)=\left\{ \begin{array}{c} \frac{\left[1+\log\left(\alpha \right)G\left(x\right)\right]}{\alpha }\;\alpha^{G\left(x\right)}\;g\left(x\right)\;if\;\alpha \neq 1,\alpha >0\\ g\left(x\right)\;if\;\alpha =1\; \end{array}\right.$$

and

(2) $$F\left(x\right)=\left\{ \begin{array}{c} \frac{{G\left(x\right)\alpha }^{G\left(x\right)}\;}{\alpha }\;if\;\alpha \neq 1,\alpha >0\\ G\left(x\right)\;if\;\alpha =1 \end{array}\right.$$

Using the same generalized family [37] presented a new extension of power Lindley distribution and utilized it to analyze wind speed data.

The key purpose of the contemporary study was to present a new four-parameter Lomax distribution. The new model is named “New Alpha Power Transformed Power Lomax (NAPTPLx) distribution”. Derived some of its important mathematical properties. The parameters of NAPTPLx distribution are estimated using the maximum likelihood (ML) estimation technique. A comprehensive simulation study is used to assess the behavior of derived ML estimators. We utilized two data sets from different fields to show the usefulness of the new distribution. In the end, we also analyze the data sets using the Bayesian estimation approach and using the Markov Chain Monte Carlo (MCMC) approach.

The remaining portions of the manuscript are organized as follows: The new probability distribution is introduced in Section 2. Quantile function, raw moments, incomplete moments, moment-generating function, order statistics, survival and hazard function, and mean residual life function are some of the mathematical properties that Section 3 derived. In Section 4, the model's parameters are estimated. In Section 5, a thorough simulation analysis is presented. Section 5 provides the formulation of the Bayesian model. Section 6 provides two examples of practical data applications. Bayesian estimation using the MCMC approach is also discussed in Section 6. In the end, we conclude our study in Section 7.

## 2 The NAPTPLx model

The cdf of the NAPTPLx distribution can be derived by incorporating equation (1) in equation (2), which is given by

$$F\left(x\right)=\frac{\left(1-\lambda^{\theta }{\left(\lambda +x^{\beta }\right)}^{-\theta }\right)\alpha^{\left(1-\lambda^{\theta }{\left(\lambda +x^{\beta }\right)}^{-\theta }\right)}}{\alpha },\alpha \neq 1,\alpha ,\beta ,\lambda >0,x>0$$

The corresponding pdf is

(3) $$f\left(x\right)=\frac{\theta \beta \lambda^{\theta }}{\alpha }x^{\beta -1}{\left(\lambda +x^{\beta }\right)}^{-\theta -1}\alpha^{1-\lambda^{\theta }{\left(\lambda +x^{\beta }\right)}^{-\theta }}\left[1+\log\left(\alpha \right)\left(1-\lambda^{\theta }{\left(\lambda +x^{\beta }\right)}^{-\theta }\right)\right]\text{.}$$

In addition, by using expansion $\alpha^{v}=\sum_{i=0}^{\infty }\frac{{\left(\log\left(\alpha \right)\right)}^{i}}{i!}v^{i}$ the alternative form of equation (3) is written as

(4) $$f\left(x\right)=\frac{\theta \beta }{\lambda }x^{\beta -1}\sum_{k=0}^{\infty }\frac{{\left(-\log\left(\alpha \right)\right)}^{k}}{k!}{\left(1+{\frac{x}{\lambda }}^{\beta }\right)}^{-\theta k-\theta -1}\left[1+\log\left(\alpha \right)\left(1-{\left(1+{\frac{x}{\lambda }}^{\beta }\right)}^{-\theta }\right)\right]\text{.}$$

The shape of the density function is based on four parameters. The limiting behavior of the NAPTPLx distribution at the lower limit (x→0) is

$$\lim_{x\to 0}f\left(x\right)=\left\{ \begin{array}{c} \infty \beta <1\\ \frac{\theta }{\alpha \lambda }\;\beta =1\\ 0\;\beta >1 \end{array}\right.$$

and the behavior of density at upper limits (x=∞) is $\lim_{x\to \infty }f\left(x\right)=0$.

**Fig. 1:**
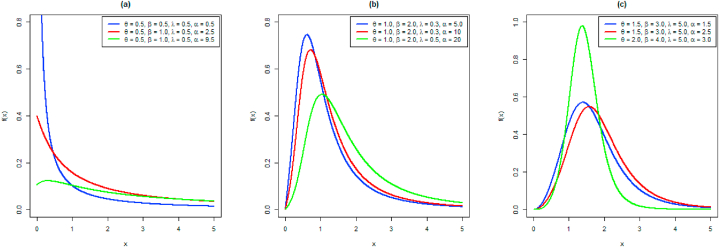
Density shapes for different choices of parameters.

Fig. 1 lists all possible shapes of the probability density function.

The adaptability of the NAPTPLx distribution with varying shape behavior is demonstrated by a pdf curve. The understudy model is categorized into three subfamilies. The pdf curves exhibit a sharp decline in responsiveness for β<1. In the second subfamily when β=1, the density curves demonstrate decreasing behavior but this subfamily has a specific initiation on the verticle axis. The pdf curves show unimodal behavior in the third subfamily. All these subfamilies with variable shapes of density curves show the flexibility of the proposed distribution.

The survival and failure rate (hazard rate) function of NAPTPLx distribution are given below

$$S\left(x\right)=1-\alpha^{-\lambda^{\theta }{\left(\lambda +x^{\beta }\right)}^{-\theta }}\left(1-\lambda^{\theta }{\left(\lambda +x^{\beta }\right)}^{-\theta }\right)\text{,}$$

and

$$h\left(x\right)=\frac{\theta \beta \lambda^{\theta }x^{\beta -1}{\left(\lambda +x^{\beta }\right)}^{-\theta -1}\alpha^{1-\lambda^{\theta }{\left(\lambda +x^{\beta }\right)}^{-\theta }}\left[1+\log\left(\alpha \right)\left(1-\lambda^{\theta }{\left(\lambda +x^{\beta }\right)}^{-\theta }\right))\right]}{\alpha \left(1-\alpha^{-\lambda^{\theta }{\left(\lambda +x^{\beta }\right)}^{-\theta }}\left(1-\lambda^{\theta }{\left(\lambda +x^{\beta }\right)}^{-\theta }\right)\right)}\text{.}$$

The cumulative hrf of the NAPTPLx distribution is

$$H\left(x\right)=-\log\left(1-\alpha^{-\lambda^{\theta }{\left(\lambda +x^{\beta }\right)}^{-\theta }}\left(1-\lambda^{\theta }{\left(\lambda +x^{\beta }\right)}^{-\theta }\right)\right)\text{.}$$

The reverse hrf of NAPTPLx distribution is

$$r\left(x\right)=\frac{\theta \beta \lambda^{\theta }x^{\beta ‐1}{\left(\lambda +x^{\beta }\right)}^{‐\theta ‐1}\alpha^{1‐\lambda^{\theta }{\left(\lambda +x^{\beta }\right)}^{‐\theta }}\left[1+\log\;\alpha \left(1‐\lambda^{\theta }{\left(\lambda +x^{\beta }\right)}^{‐\theta }\right)\right]}{\left(1‐\lambda^{\theta }{\left(\lambda +x^{\beta }\right)}^{‐\theta }\right)\alpha^{\left(1‐\lambda^{\theta }{\left(\lambda +x^{\beta }\right)}^{‐\theta }\right)}}$$

**Fig. 2:**
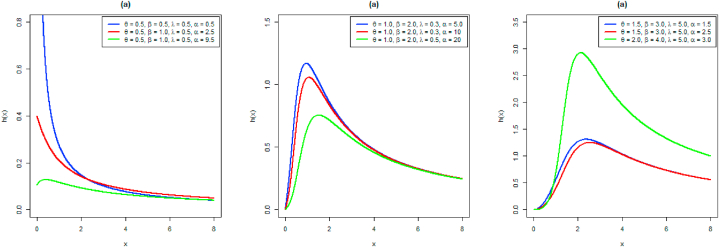
hrf plots for different parameter combinations.

The different shapes of the hrf are given in Fig. 2.

Fig. 2 indicates some interesting results about the failure rate pattern NAPTPLx distribution. The hrf curves starting from the origin when β>1, start from some specific point at the y-axis for β=1 and the graph is L-shaped for β<1.

## 3 Statistical properties

In this section, we derived several essential statistical features for the NAPTPLx distribution.

### 3.1 Quantiles

The quantile function (qf) is a key statistical measure and is utilized for data generation. The qf of the NAPTPLx distribution is given in equation (5)

(5) $$\left(1-\lambda^{\theta }{\left(\lambda +x^{\beta }\right)}^{-\theta }\right)\alpha^{\left(1-\lambda^{\theta }{\left(\lambda +x^{\beta }\right)}^{-\theta }\right)}=\alpha p,0<p<1\text{.}$$

where p follows a uniform distribution. The quartiles of the NAPTPLx distribution can be calculated numerically and presented in Table 1.

**Table 1:** First, second, and third quartile values for some selected values of parameters.

Parameters				Q_1_	Median	Q_3_
θ	β	λ	α			
0.5	0.5	0.5	0.5	0.029628	0.302408	3.810631
0.5	1.0	0.5	2.5	1.006260	4.205799	23.57233
0.5	2.0	0.5	9.5	2.727576	11.32462	65.19728
1.0	2.0	0.3	5.0	0.583314	0.946022	1.575373
1.0	2.0	0.3	10.0	0.687206	1.086392	1.786488
1.0	2.0	0.5	20.0	1.008958	1.565673	2.551431
1.5	3.0	5.0	1.5	1.149493	1.595928	2.163814
1.5	3.0	5.0	2.5	1.304768	1.771210	2.362701
1.5	3.0	5.0	3.0	1.153872	1.421891	1.719237

### 3.2 Moments

Moments play a significant part in statistical analysis and its applications. It may be utilized to investigate the utmost prominent traits and properties of a model, such as mean, variance, dispersion index, skewness, and kurtosis.

The rth moments can be derived using the following formula

(6) $$\mu_{r}^{\prime }=\int_{0}^{\infty }x^{r}f\left(x\right)dx\text{.}$$

Now substituting equation (4) into equation (6)

$$\mu_{r}^{\prime }=\frac{\theta \beta }{\lambda }\int_{0}^{\infty }x^{r+\beta -1}\sum_{k=0}^{\infty }\frac{{\left(-\log\left(\alpha \right)\right)}^{k}}{k!}{\left(1+{\frac{x}{\lambda }}^{\beta }\right)}^{-\theta k-\theta -1}\left[1+\log\left(\alpha \right)\left(1-{\left(1+{\frac{x}{\lambda }}^{\beta }\right)}^{-\theta }\right)\right]dx\text{,}$$

(7) $$=\frac{\theta \beta }{\lambda }\sum_{k=0}^{\infty }\frac{{\left(-\log\left(\alpha \right)\right)}^{k}}{k!}\int_{0}^{\infty }x^{r+\beta -1}{\left(1+{\frac{x}{\lambda }}^{\beta }\right)}^{-\theta k-\theta -1}dx-\frac{\theta \beta }{\lambda }\sum_{k=0}^{\infty }\frac{{\left(-\log\left(\alpha \right)\right)}^{k}}{k!}\log\left(\alpha \right)\int_{0}^{\infty }x^{r+\beta -1}{\left(1+{\frac{x}{\lambda }}^{\beta }\right)}^{-\theta k-\theta -1}dx\text{,}$$

Making transformation $y={\frac{x}{\lambda }}^{\beta }$, we get

(8) $$\mu_{r}^{\prime }=\theta \lambda^{\frac{r}{\beta }}\sum_{k=0}^{\infty }\frac{{\left(-\log\left(\alpha \right)\right)}^{k}}{k!}\int_{0}^{\infty }y^{\frac{r}{\beta }}{\left(1+y\right)}^{-\theta k-\theta -1}dy-\theta \lambda^{\frac{r}{\beta }}\sum_{k=0}^{\infty }\frac{{\left(-\log\left(\alpha \right)\right)}^{k}}{k!}\log\left(\alpha \right)\int_{0}^{\infty }y^{\frac{r}{\beta }}{\left(1+y\right)}^{-\theta k-\theta -1}dy\text{,}$$

Again, transforming $y=\frac{w}{1-w}$ in equation (8), and after some simplifications we get the following expression

$$\mu_{r}^{\prime }=\theta \lambda^{\frac{r}{\beta }}\sum_{k=0}^{\infty }\frac{{\left(-\log\left(\alpha \right)\right)}^{k}}{k!}Β\left(\frac{r}{\beta }+1,\theta \left(k+1\right)-\frac{r}{\beta }\right)-\theta \lambda^{\frac{r}{\beta }}\sum_{k=0}^{\infty }\frac{{\left(-\log\left(\alpha \right)\right)}^{k}}{k!}\log\left(\alpha \right)Β\left(\frac{r}{\beta }+1,\theta \left(k+2\right)-\frac{r}{\beta }\right)\text{.}$$

where B(.,.) stands for beta function.

### 3.3 Moment-generating function

Moment generating function (mgf) can be gained via the following formula

$$M_{X}\left(t\right)=E\left(e^{tx}\right)=\int_{0}^{\infty }e^{tx}f\left(x\right)dx=\sum_{r=0}^{\infty }\frac{{\left(t\right)}^{r}}{r!}E\left(x^{r}\right)$$

The mgf of NAPTPLx distribution is derived by using equation (4)

$$M_{X}\left(t\right)=\sum_{r=0}^{\infty }\frac{{\left(t\right)}^{r}}{r!}\left[\theta \lambda^{\frac{r}{\beta }}\sum_{k=0}^{\infty }\frac{{\left(-\log\left(\alpha \right)\right)}^{k}}{k!}Β\left(\frac{r}{\beta }+1,\theta \left(k+1\right)-\frac{r}{\beta }\right)-\theta \lambda^{\frac{r}{\beta }}\sum_{k=0}^{\infty }\frac{{\left(-\log\left(\alpha \right)\right)}^{k}}{k!}\log\left(\alpha \right)Β\left(\frac{r}{\beta }+1,\theta \left(k+2\right)-\frac{r}{\beta }\right)\right]\text{.}$$

Table 2 lists some computational metrics for various parameter selections, including mean, standard deviation (SD), coefficient of variation (CV), dispersion index (DI), coefficient of skewness (CS), and kurtosis (CK).

**Table 2:** Skewness, mean, kurtosis, and variance values.

Parameters				Mean	SD	CV	DI	CS	CK
θ	β	λ	α						
0.5	0.5	0.5	0.5	94.1578	613.355	6.51412	3995.47	10.0404	118.691
0.5	1.0	0.5	2.5	128.122	654.630	5.10944	3344.79	8.94604	96.9537
0.5	2.0	0.5	9.5	208.656	836.167	4.00740	3350.85	6.89781	58.3031
1.0	2.0	0.3	5.0	1.44299	3.44232	2.38554	8.21179	382.492	556884.0
1.0	2.0	0.3	10.0	1.64544	3.83873	2.33295	8.95557	348.923	455674.4
1.0	2.0	0.5	20.0	2.36055	5.31679	2.25235	11.9753	264.360	249546.0
1.5	3.0	5.0	1.5	1.76758	0.96342	0.54505	0.52511	2.81028	48.1216
1.5	3.0	5.0	2.5	1.94878	1.01803	0.52239	0.53181	2.85410	50.1831
1.5	3.0	5.0	3.0	1.46304	0.47171	0.32242	0.15208	0.91904	6.27517

### 3.4 Incomplete moments

Incomplete moments of NAPTPLx distribution are

$$\varphi \left(x\right)=\int_{0}^{x}v^{r}f\left(v\right)dv$$

$$=\frac{\theta \beta }{\lambda }\int_{0}^{x}x^{r+\beta -1}\sum_{k=0}^{\infty }\frac{{\left(-\log\left(\alpha \right)\right)}^{k}}{k!}{\left(1+{\frac{x}{\lambda }}^{\beta }\right)}^{-\theta k-\theta -1}\left[1+\log\left(\alpha \right)\left(1-{\left(1+{\frac{x}{\lambda }}^{\beta }\right)}^{-\theta }\right)\right]dx\text{,}$$

and

$$\varphi \left(x\right)=\theta \lambda^{\frac{r}{\beta }}\sum_{k=0}^{\infty }\frac{{\left(-\log\left(\alpha \right)\right)}^{k}}{k!}Β\left(\frac{r}{\beta }+1,\theta \left(k+1\right)-\frac{r}{\beta }\right)-\theta \lambda^{\frac{r}{\beta }}\sum_{k=0}^{\infty }\frac{{\left(-\log\left(\alpha \right)\right)}^{k}}{k!}\log\left(\alpha \right)Β\left(\frac{r}{\beta }+1,\theta \left(k+2\right)-\frac{r}{\beta }\right)\text{.}$$

### 3.5 Order statistics

Let $X_{1},X_{2},X_{3},\ldots ,X_{n}$ be a random sample taken from the NAPTPLx distribution and $X_{1:n},X_{2:n},X_{3:n},\ldots ,X_{n:n}$ be the corresponding order statistics. The probability density function of kth-order statistics is

(9) $$f_{k:n}\left(x\right)=\frac{n!}{\left(k-1\right)!\left(n-k\right)!}f\left(x\right){\left[F\left(x\right)\right]}^{k-1}{\left[1-F\left(x\right)\right]}^{n-k}$$

where f(x) and F(x) are pdf and cdf of NAPTPLx distribution. We can use the binomial expansion of ${\left[1-F\left(x\right)\right]}^{n-k}$ given as follows

(10) $${\left[1-F\left(x\right)\right]}^{n-k}=\sum_{u=0}^{n-k}{\left(-1\right)}^{u}\left(\begin{array}{c} n-k\\ u \end{array}\right)F{\left(x\right)}^{u}$$

Putting equation (10) in equation (9)

$$f_{k:n}\left(x\right)=\frac{n!}{\left(k-1\right)!\left(n-k\right)!}\sum_{u=0}^{n-k}{\left(-1\right)}^{u}\left(\begin{array}{c} n-k\\ u \end{array}\right)f\left(x\right)F{\left(x\right)}^{k+u-1}$$

The kth order statistics for NAPTPLx distribution can be expressed as

(11) $$f_{k:n}\left(x\right)=\frac{n!}{\left(k-1\right)!\left(n-k\right)!}\sum_{u=0}^{n-k}{\left(-1\right)}^{u}\left(\begin{array}{c} n-k\\ u \end{array}\right)\left(\frac{\theta \beta \lambda^{\theta }x^{\beta -1}}{\alpha {\left(\lambda +x^{\beta }\right)}^{\theta +1}}\alpha^{1-\lambda^{\theta }{\left(\lambda +x^{\beta }\right)}^{-\theta }}\left[1+\left(1-\lambda^{\theta }{\left(\lambda +x^{\beta }\right)}^{-\theta }\right)\log\left(\alpha \right)\right]\right)\times {\left(\frac{\left(1-\lambda^{\theta }{\left(\lambda +x^{\beta }\right)}^{-\theta }\right)\alpha^{\left(1-\lambda^{\theta }{\left(\lambda +x^{\beta }\right)}^{-\theta }\right)}}{\alpha }\right)}^{k+u-1}$$

when r=1 and r=n in equation (11), we can obtain the expressions of smallest and largest order statistics, respectively.

### 3.6 Mean residual life

The mean residual life (MRL) of the NAPTPLx distribution is derived as

$$m\left(t\right)=\frac{1}{R\left(t\right)}\int_{t}^{\infty }xf\left(x\right)dx-t$$

Now solve an integral part

$$\int_{t}^{\infty }xf\left(x\right)dx=\frac{\theta \beta }{\lambda }\int_{t}^{\infty }x^{\beta }\sum_{k=0}^{\infty }\frac{{\left(-\log\left(\alpha \right)\right)}^{k}}{k!}{\left(1+{\frac{x}{\lambda }}^{\beta }\right)}^{-\theta k-\theta -1}\left[1+\log\left(\alpha \right)\left(1-{\left(1+{\frac{x}{\lambda }}^{\beta }\right)}^{-\theta }\right)\right]dx$$

$$\int_{t}^{\infty }xf\left(x\right)dx=\frac{\theta \beta }{\lambda }\sum_{k=0}^{\infty }\frac{{\left(-\log\left(\alpha \right)\right)}^{k}}{k!}\left(1+\log\left(\alpha \right)\right)\int_{t}^{\infty }x^{\beta }{\left(1+{\frac{x}{\lambda }}^{\beta }\right)}^{-\theta k-\theta -1}dx-\frac{\theta \beta }{\lambda }\sum_{k=0}^{\infty }\frac{{\left(-\log\left(\alpha \right)\right)}^{k}}{k!}\log\left(\alpha \right)\int_{t}^{\infty }x^{\beta }{\left(1+{\frac{x}{\lambda }}^{\beta }\right)}^{-\theta k-2\theta -1}dx$$

Making transformation and algebraic simplification, we get

(12) $$\int_{t}^{\infty }xf\left(x\right)dx=\theta \lambda^{\frac{1}{\beta }}\left(1+\log\left(\alpha \right)\right)\sum_{k=0}^{\infty }\frac{{\left(-\log\left(\alpha \right)\right)}^{k}}{k!}Β\left(\frac{1}{\beta }+1,\theta \left(k+1\right)-\frac{1}{\beta }\right)-\theta \lambda^{\frac{1}{\beta }}\sum_{k=0}^{\infty }\frac{{\left(-\log\left(\alpha \right)\right)}^{k}}{k!}\log\left(\alpha \right)Β\left(\frac{1}{\beta }+1,\theta \left(k+2\right)-\frac{1}{\beta }\right)$$

Substituting equation (12) in equation (11), we get

$$m\left(t\right)=\frac{1}{R\left(t\right)}\left[\theta \lambda^{\frac{1}{\beta }}\left(1+\log\left(\alpha \right)\right)\sum_{k=0}^{\infty }\frac{{\left(-\log\left(\alpha \right)\right)}^{k}}{k!}Β\left(\frac{1}{\beta }+1,\theta \left(k+1\right)-\frac{1}{\beta }\right)-\theta \lambda^{\frac{1}{\beta }}\sum_{k=0}^{\infty }\frac{{\left(-\log\left(\alpha \right)\right)}^{k}}{k!}\log\left(\alpha \right)Β\left(\frac{1}{\beta }+1,\theta \left(k+2\right)-\frac{1}{\beta }\right)\right]-t\text{.}$$

## 4 Parameter estimation of NAPTPLx distribution

In this section, we consider maximum likelihood estimation (MLE) for a given sample of size from the NAPTPLx distribution. Its relative log-likelihood function is

$$l=n\;\log\left(\theta \right)+n\;\log\left(\beta \right)+n\theta \;\log\left(\lambda \right)-n\;\log\left(\alpha \right)+\left(\beta -1\right)\sum_{i=1}^{n}\log\left(x_{i}\right)-\left(\theta +1\right)\sum_{i=1}^{n}\log\left(\lambda +x_{i}^{\beta }\right)+\log\left(\alpha \right)\sum_{i=1}^{n}\left[1-\lambda^{\theta }{\left(\lambda +x_{i}^{\beta }\right)}^{-\theta }\right]+\sum_{i=1}^{n}\log\left(1+\log\left(\alpha \right)\left(1-\lambda^{\theta }{\left(\lambda +x_{i}^{\beta }\right)}^{-\theta }\right)\right)\text{.}$$

The partial derivatives are written as follows:

$$\frac{\partial l}{\partial \alpha }=-\frac{n}{\alpha }+\frac{1}{\alpha }\sum_{i=1}^{n}\left[1-\lambda^{\theta }{\left(\lambda +x_{i}^{\beta }\right)}^{-\theta }\right]+\sum_{i=1}^{n}\frac{\left[1-\lambda^{\theta }{\left(\lambda +x_{i}^{\beta }\right)}^{-\theta }\right]}{\alpha \left[1+\log\left(\alpha \right)\left(1-\lambda^{\theta }{\left(\lambda +x_{i}^{\beta }\right)}^{-\theta }\right)\right]}\text{,}$$

$$\frac{\partial l}{\partial \beta }=\frac{n}{\beta }+\sum_{i=1}^{n}\log\left(x_{i}\right)-\left(\theta +1\right)\sum_{i=1}^{n}\frac{x_{i}^{\beta }\;\log\left(x_{i}\right)}{\lambda +x_{i}^{\beta }}+\theta \lambda^{\theta }\;\log\left(\alpha \right)\sum_{i=1}^{n}x_{i}^{\beta }\;\log\left(x_{i}\right){\left(\lambda +x_{i}^{\beta }\right)}^{-\theta -1}+\theta \lambda^{\theta }\;\log\left(\alpha \right)\sum_{i=1}^{n}\frac{x_{i}^{\beta }\;\log\left(x_{i}\right){\left(\lambda +x_{i}^{\beta }\right)}^{-\theta -1}}{1+\log\left(\alpha \right)\left(1-\lambda^{\theta }{\left(\lambda +x_{i}^{\beta }\right)}^{-\theta }\right)}\text{,}$$

$$\frac{\partial l}{\partial \theta }=\frac{n}{\theta }+n\;\log\left(\lambda \right)-\sum_{i=1}^{n}\log\left(\lambda +x_{i}^{\beta }\right)-\log\left(\alpha \right)\sum_{i=1}^{n}\left[\lambda^{\theta }{\left(\lambda +x_{i}^{\beta }\right)}^{-\theta }\left(\log\left(\lambda \right)+{\log\left(\lambda +x_{i}^{\beta }\right)}^{-\theta }\right)\right]+\log\left(\alpha \right)\sum_{i=1}^{n}\frac{\left(\lambda^{\theta }{\left(\lambda +x_{i}^{\beta }\right)}^{-\theta }\left(-\log\left(\lambda \right)+{\log\left(\lambda +x_{i}^{\beta }\right)}^{-\theta }\right)\right)}{\left[1+\log\left(\alpha \right)\left(1-\lambda^{\theta }{\left(\lambda +x_{i}^{\beta }\right)}^{-\theta }\right)\right]}\text{,}$$

and

$$\frac{\partial l}{\partial \lambda }=\frac{n\theta }{\lambda }-\sum_{i=1}^{n}\frac{\left(\theta +1\right)}{\left(\lambda +x_{i}^{\beta }\right)}-\log\left(\alpha \right)\sum_{i=1}^{n}\frac{\theta \lambda^{\theta }x_{i}^{\beta }{\left(\lambda +x_{i}^{\beta }\right)}^{-\theta }}{\lambda \left(\lambda +x_{i}^{\beta }\right)}-\log\left(\alpha \right)\sum_{i=1}^{n}\frac{\left(\theta \lambda^{\theta }{\left(\lambda +x_{i}^{\beta }\right)}^{-\theta }\right)}{1+\log\left(\alpha \right)\left(1-\lambda^{\theta }{\left(\lambda +x_{i}^{\beta }\right)}^{-\theta }\right)\;}\left[\frac{x_{i}^{\beta }}{\lambda \left(\lambda +x_{i}^{\beta }\right)}\right]\text{.}$$

Since the above-derived estimators cannot be solved precisely, we will utilize R software to solve these non-linear equations using Newton-Raphson and other optimization techniques.

## 5 Simulation study

A complete simulation analysis is used in this section to test the behavior of derived maximum likelihood estimators. Random samples of 10, 30, 80, 100, and 200 are created for this numerical process. The process is performed 10,000 times. Absolute bias (AB) and mean square error (MSE) are obtained and used for evaluation. The following combination of parameters is utilized to simulate samples. The simulation results are given in Table 3, Table 4.

**Table 3:** Simulation study results for the NAPTPLx distribution.

Par.	n	λ=0.1,α=0.2,θ=0.5,β=0.5			λ=0.5,α=0.1,θ=1.5,β=0.7		
		AE	AB	MSE	AE	AB	MSE
$\hat{\alpha }$	10	0.5000	0.3000	0.2250	0.1980	0.0980	0.2490
	30	0.4290	0.2290	0.1855	0.1880	0.0880	0.2440
	80	0.3830	0.1830	0.1085	0.1700	0.0700	0.2350
	100	0.2335	0.0335	0.0833	0.1520	0.0520	0.2260
	200	0.2261	0.0261	0.0320	0.1260	0.0260	0.2130
$\hat{\beta }$	10	0.6059	0.1059	0.0293	0.9067	0.2067	0.0565
	30	0.5616	0.0616	0.0149	0.8343	0.1343	0.0430
	80	0.5295	0.0295	0.0057	0.7816	0.0816	0.0280
	100	0.5195	0.0195	0.0044	0.7752	0.0752	0.0281
	200	0.5081	0.0081	0.0012	0.7141	0.0141	0.0195
$\hat{\theta }$	10	0.6260	0.1260	0.9177	2.3768	0.8768	0.9550
	30	0.5727	0.0727	0.2967	2.3603	0.8603	0.3199
	80	0.5274	0.0274	0.0281	2.3597	0.8597	0.1973
	100	0.5162	0.0162	0.0198	2.1862	0.6862	0.1818
	200	0.5099	0.0099	0.0047	1.9739	0.4739	0.1068
$\hat{\lambda }$	10	0.4053	0.3053	0.0484	0.6619	0.1619	0.3090
	30	0.3901	0.2901	0.0396	0.5823	0.0823	0.0554
	80	0.3372	0.2372	0.0183	0.5776	0.0776	0.0414
	100	0.2545	0.1545	0.0107	0.5689	0.0689	0.0392
	200	0.1871	0.0871	0.0023	0.5208	0.0208	0.0207

**Table 4:** Simulation study results for NAPTPLx distribution.

Par.	n	λ=0.5,α=0.2,θ=0.5,β=0.5			λ=0.5,α=0.2,θ=1.5,β=0.5		
		AE	AB	MSE	AE	AB	MSE
$\hat{\alpha }$	10	0.3900	0.1900	0.2450	0.3800	0.1800	0.2401
	30	0.3700	0.1700	0.2351	0.3300	0.1300	0.2153
	80	0.3449	0.1449	0.2227	0.2800	0.0800	0.1556
	100	0.3198	0.1198	0.2102	0.2149	0.0149	0.1530
	200	0.2345	0.0345	0.1676	0.2091	0.0091	0.0604
$\hat{\beta }$	10	0.7123	0.2123	0.0956	0.6617	0.1617	0.0533
	30	0.7075	0.2075	0.0614	0.6160	0.1160	0.0259
	80	0.6887	0.1887	0.0570	0.5779	0.0779	0.0137
	100	0.6671	0.1671	0.0605	0.5714	0.0714	0.0102
	200	0.6209	0.1209	0.0385	0.5283	0.0283	0.0038
$\hat{\theta }$	10	0.9344	0.4344	0.1300	4.2976	2.7976	0.1302
	30	0.8471	0.3471	0.1318	2.6122	1.1122	0.0992
	80	0.7945	0.2945	0.1015	2.5601	1.0601	0.0353
	100	0.7599	0.2599	0.0845	2.5314	1.0314	0.0274
	200	0.6878	0.1878	0.0573	2.4916	0.9916	0.0065
$\hat{\lambda }$	10	0.6332	0.1332	0.0641	1.5478	1.0478	0.5533
	30	0.5868	0.0868	0.0495	0.6335	0.1335	0.4779
	80	0.5831	0.0831	0.0506	0.5970	0.0970	0.0266
	100	0.5774	0.0774	0.0564	0.5731	0.0731	0.0189
	200	0.5562	0.0562	0.0415	0.5265	0.0265	0.0054

## 6 Bayesian analysis

Since the Bayesian framework treats model parameters as random variables, their prior distributions must be specified to estimate the NAPTPLx distribution's parameters. Therefore, choosing a prior distribution is crucial for estimating parameters. The independent gamma distributions $G\left(a_{1},b_{1}\right)$, $G\left(a_{2},b_{2}\right)$, $G\left(a_{3},b_{3}\right)$ and $G\left(a_{4},b_{4}\right)$ are our choice for α, β, θ, and λ prior distributions. Gamma prior is flexible with a non-informative domain, and it also provides conjugate prior for the likelihood function. These factors led to the selection of this prior density. The densities of the proposed gamma distributions are as follows:

$$p\left(\alpha \right)=\frac{b_{1}^{a_{1}}}{\Gamma \left(a_{1}\right)}\alpha^{a_{1}-1}e^{-b_{1}\alpha },\alpha >0\text{,}$$

$$p\left(\beta \right)=\frac{b_{2}^{a_{2}}}{\Gamma \left(a_{2}\right)}\beta^{a_{2}-1}e^{-b_{2}\alpha },\beta >0\text{,}$$

$$p\left(\theta \right)=\frac{b_{3}^{a_{3}}}{\Gamma \left(a_{3}\right)}\theta^{a_{3}-1}e^{-b_{3}\theta },\theta >0\text{,}$$

$$p\left(\lambda \right)=\frac{b_{4}^{a_{4}}}{\Gamma \left(a_{4}\right)}\lambda^{a_{4}-1}e^{-b_{4}\lambda },\lambda >0\text{,}$$

where $a_{1}$, $a_{2}$*,* $a_{3}$*,* $a_{4}$*,* $b_{1}$, $b_{2}$*,* $b_{3}$*,* and $b_{4}$ are the hyperparameters of the prior distribution and all are positive constants.

Hence, we have

p(α,β,θ,λ)=p(α)p(β)p(θ)p(λ)

The posterior density function is proportional to the product of the likelihood function given in the previous section and the joint prior distribution for these parameters. The expression can be written as

$$p\left(\alpha ,\beta ,\theta ,\lambda |x\right)\propto \alpha^{a_{1}+n-1}\beta^{a_{2}+n-1}\theta^{a_{3}+n-1}\lambda^{a_{4}+n-1}e^{-\left(b_{1}\alpha \mp \beta +b_{3}\theta +b_{4}\lambda \right)}L_{1}$$

where $L_{1}$ is the log-likelihood function.

There are no closed-form inferences because the posterior density is convoluted. Therefore, suggest simulating samples from the posterior utilizing MCMC approaches, especially Gibbs sampling and Metropolis-Hastings (M − H) algorithms, enabling straightforward sample-based conclusions. The M − H algorithm is available in MCMCpack, an R package that contains functions to perform Bayesian analysis. The model was run for 1,000,000 iterations, with a burn-in phase of 10,000 simulated samples and a size 200 thinning interval.

For all parameters of interest, the highest posterior density intervals (abbreviated as HPD intervals or HDI) with 95% coverage were obtained. The shortest interval among all Bayesian credible intervals is an HPD interval. Convergence was evaluated visually using traceplots from each MCMC chain and quantitatively using the Geweke criterion. The Geweke's z-score is generated by the difference between the two-sample means divided by its estimated standard error, assuming asymptotic independence between these two components. This statistic has an asymptotically standard normal distribution if the MCMC samples are selected from a stationary distribution.

## 7 Data analysis

In this section, two data sets from different fields are utilized to illustrate the applicability of the proposed NAPTPLx distribution. The distributions used for comparison purposes are the Lomax (Lx) distribution, the Power Lomax (PLx) distribution, the Marshal Olkin Lomax (MOLx) distribution, and the Marshal Olkin Power Lomax (MOPLx) distribution. The density functions of these models are given in Appendix. The parameters of all considered competitive distributions are estimated using MLEs. Some renowned model selection and goodness-of-fit measures such as the AIC (Akaike Information Criterion), BIC (Bayesian Information Criterion), (AD) Anderson Darling, (CVM) Cramer von Misses, and (KS) Kolmogorov-Smirnov statistic are used to identify the best-fitted distribution. The model has maximum log-likelihood values, p-values of AD, CVM, and KS, and minimum values of AIC, BIC, and AD, CVM, and KS statistic values that were chosen as the best fits for the data.

### 7.1 Analysis of bladder cancer patients’ data

The first data set contains the remission time of 128 bladder cancer patients (monthly) data. It was originally studied by Ref. [38]. The observations are; 0.08, 2.09, 3.48, 4.87, 6.94, 8.66, 13.11, 23.63, 0.20, 2.23, 3.52, 4.98, 6.97, 9.02, 13.29, 0.40, 2.26, 3.57, 5.06, 7.09, 9.22, 13.80, 25.74, 0.50, 2.46, 3.64, 5.09, 7.26, 9.47, 14.24, 25.82, 0.51, 2.54, 3.70, 5.17, 7.28, 9.74, 14.76, 26.31, 0.81, 2.62, 3.82, 5.32, 7.32, 10.06, 14.77, 32.15, 2.64, 3.88, 5.32, 7.39, 10.34, 14.83, 34.26, 0.90, 2.69, 4.18, 5.34, 7.59, 10.66, 15.96, 36.66, 1.05, 2.69, 4.23, 5.41, 7.62, 10.75, 16.62, 43.01, 1.19, 2.75, 4.26, 5.41, 7.63, 17.12, 46.12, 1.26, 2.83, 4.33, 5.49, 7.66, 11.25, 17.14, 79.05, 1.35, 2.87, 5.62, 7.87, 11.64, 17.36, 1.40, 3.02, 4.34, 5.71, 7.93, 11.79, 18.10, 1.46, 4.40, 5.85, 8.26, 11.98, 19.13, 1.76, 3.25, 4.50, 6.25, 8.37, 12.02, 2.02, 3.31, 4.51, 6.54, 8.53, 12.03, 20.28, 2.02, 3.36, 6.76, 12.07, 21.73, 2.07, 3.36, 6.93, 8.65, 12.63, and 22.69.

**Fig. 3:**
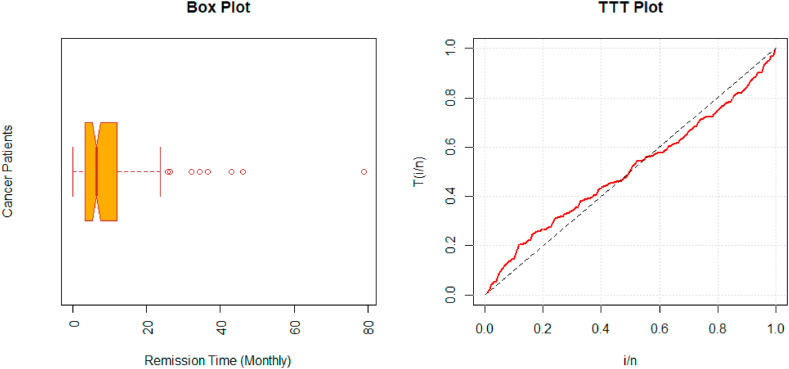
Boxplot (left) and TTT plot (right) of bladder cancer patient's data.

The nature of this data set is assessed using a boxplot. We also plot the Total Time on Test (TTT) plot to identify the failure rate pattern of the considered data set. The boxplots and TTT plots are given in Fig. 3. The MLEs with standard errors (SE) for each model and goodness of fit measures for the first data set are given in Table 5.

**Table 5:** Estimates (SE) and goodness-of-fit statistics of MLEs: The bladder cancer patient's data.

Dist.	Para.	Estimates	SE	KS	AD	CVM	−l	AIC	BIC
**Lx**	$\hat{\theta }$	13.968	15.472	0.0966	1.3761	0.2125	413.83	831.67	837.37
	$\hat{\lambda }$	121.30	143.52						
**PLx**	$\hat{\theta }$	2.0695	0.9666	0.0350	0.1216	0.0175	409.74	825.48	834.04
	$\hat{\lambda }$	34.858	13.899						
	$\hat{\beta }$	1.4277	0.1777						
**MOLx**	$\hat{\theta }$	2.2835	0.5590	0.0319	0.1082	0.0157	409.54	825.08	833.63
	$\hat{\lambda }$	2.0558	2.6124						
	$\hat{a}$	23.658	35.858						
**MOPLx**	$\hat{\theta }$	2.1413	1.8815	0.0321	0.1469	0.0189	409.81	827.63	839.04
	$\hat{\lambda }$	71.527	302.24						
	$\hat{a}$	0.5380	1.8106						
	$\hat{\beta }$	1.5076	0.3009						
**NAPTPLx**	$\hat{\theta }$	2.9503	3.1204	0.0105	0.0355	0.0105	407.40	823.80	832.21
	$\hat{\lambda }$	1.0119	0.5549						
	$\hat{a}$	9.6155	6.4639						
	$\hat{\beta }$	7.5122	18.365						

**Fig. 4:**
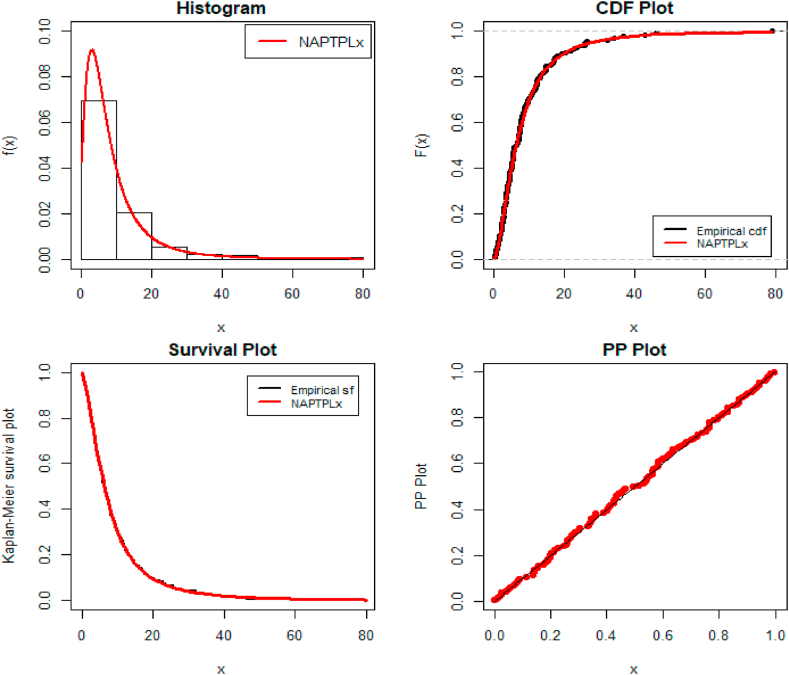
Fitted Distribution for the bladder cancer patient's data.

Fig. 4 shows the fitted density over the histogram of empirical data, the empirical cdf in the black line, and the estimated cdf in the red line. Additionally, fitted survival function and probability-probability (PP) plots of the NAPTPLx distribution for the bladder cancer patients data set.

### 7.2 Analysis of income tax data

**Fig. 5:**
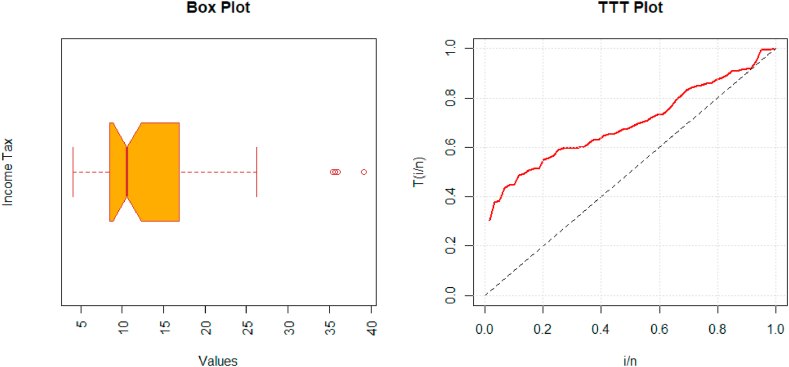
Boxplot (left) and TTT plot (right) of income tax data.

The second data set is about Egypt's monthly income tax from the duration of January 2006 to November 2010. The data observations are; 5.9, 20.4, 14.9, 16.2, 17.2, 7.8, 6.1, 9.2, 10.2, 9.6, 13.3, 8.5, 21.6, 18.5, 5.1, 6.7, 17.0, 8.6, 9.7, 39.2, 35.7, 15.7, 9.7, 10.0, 4.1, 36.0, 8.5, 8.0, 9.2, 26.2, 21.9, 16.7, 21.3, 35.4, 14.3, 8.5, 10.6, 19.1, 20.5, 7.1, 7.7, 18.1, 16.5, 11.9, 7.0, 8.6, 12.5, 10.3, 11.2, 6.1, 8.4, 11.0, 11.6, 11.9, 5.2, 6.8, 8.9, 7.1, and 10.8. The boxplot and TTT plot for the second data set are given in Fig. 5. The MLEs and goodness of fit measures are given in Table 6.

**Table 6:** Estimates (SE) and goodness-of-fit statistics of MLEs: The income tax data.

Dist.	Para.	Estimates	SE	KS	AD	CVM	−l	AIC	BIC
**Lx**	$\hat{\theta }$	98.121	142.39	0.3049	6.996	1.3286	212.70	429.41	433.56
	$\hat{\lambda }$	1313.7	1911.1						
**PLx**	$\hat{\theta }$	0.5791	0.2063	0.0740	0.3462	0.0481	189.64	385.29	393.52
	$\hat{\lambda }$	7567.1	7469.8						
	$\hat{\beta }$	4.0836	0.6335						
**MOLx**	$\hat{\theta }$	3.8466	0.6765	0.0764	0.7234	0.0985	191.89	389.79	396.02
	$\hat{\lambda }$	2.2710	1.5269						
	$\hat{a}$	999.36	1316.7						
**MOPLx**	$\hat{\theta }$	0.8091	0.3817	0.0691	0.5395	0.0788	190.51	389.03	397.34
	$\hat{\lambda }$	49.638	85.415						
	$\hat{a}$	85.237	127.46						
	$\hat{\beta }$	3.6952	1.5109						
**NAPTPLx**	$\hat{\theta }$	0.8781	1.0161	0.0632	0.2553	0.0305	187.66	384.33	391.64
	$\hat{\lambda }$	60.317	200.55						
	$\hat{a}$	1564.3	66.138						
	$\hat{\beta }$	2.8773	2.6991						

**Fig. 6:**
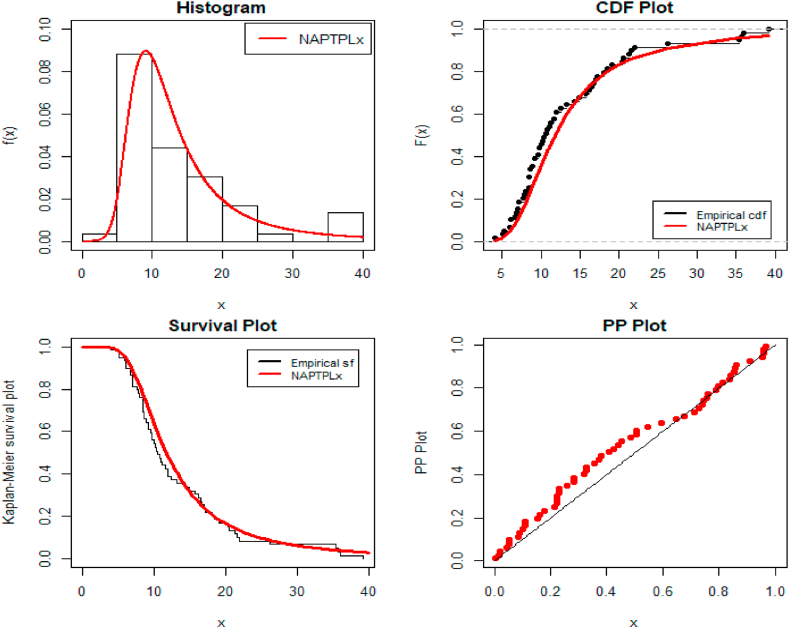
Fitted Distribution for the income tax data.

Fig. 6 shows the fitted density over the histogram of empirical data, the empirical cdf, and the estimated cdf. Additionally, fitted survival function and PP plots of the NAPTPLx distribution for the bladder cancer patients data set.

According to Table 5, Table 6, the proposed distribution has lower values and is the best fit for the competitive distributions. Fig. 4, Fig. 6 show fitted pdf, cdf, survival function, and PP plots for both the bladder cancer and monthly income tax data sets. As a result, the NAPTPLx distribution fitting improved when compared to other distributions.

### 7.3 Analysis via Bayesian method

**Fig. 7:**
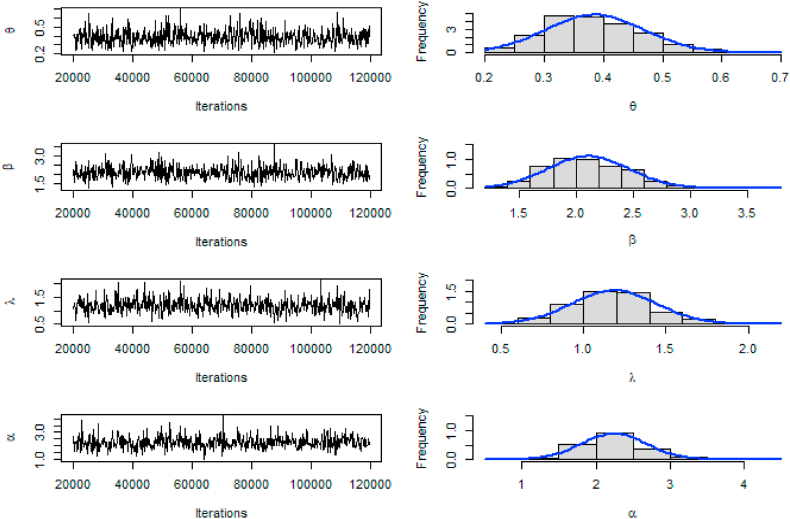
Trace and Posterior density plot based on the first data set.

**Fig. 8:**
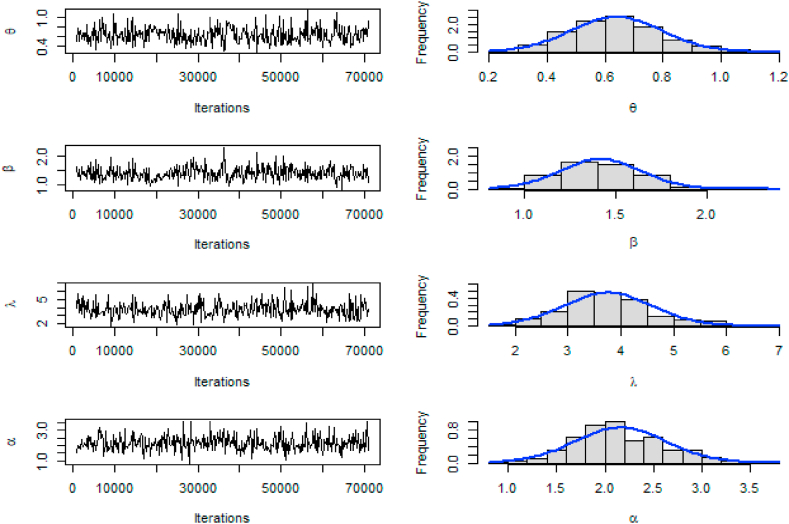
Trace and Posterior density plot based on the second data set.

Under the Bayesian methodology described in Section 5, the prior distributions of α, β, θ, and λ and were assumed to be $\alpha ∼Gamma\left(a_{1},b_{1}\right)$, $\beta ∼Gamma\left(a_{2},b_{2}\right)$, $\theta ∼Gamma\left(a_{3},b_{3}\right)$ and $\lambda ∼Gamma\left(a_{4},b_{4}\right)$. The posterior means, standard errors, Geweke's Z-score, lower and upper HPD are given in Table 7. The traceplot, and histogram of posterior density are used for the evaluation of the MCMC iterations. The posterior samples for the parameters are shown in Fig. 7, Fig. 8.

**Table 7:** Bayesian estimates, SE, HPD, and Geweke's score for both data sets.

Data		Estimates	SD	Lower	Upper	Geweke's z-score
Bladder cancer patients	θ	0.3853	0.0836	0.2257	0.5330	−0.5652
	β	2.1067	0.3620	1.5453	2.8663	−0.5168
	λ	1.1853	0.2593	0.7314	1.7640	−0.2494
	α	2.2414	0.4478	1.4220	3.2043	−1.2630
Income Tax	θ	0.6392	0.1589	0.3572	0.9558	−0.0766
	β	1.4132	0.2232	0.9951	1.8006	−0.5657
	λ	3.7532	0.8398	2.3791	5.6564	0.0107
	α	2.1588	0.4755	1.3530	3.1616	0.2018

## 8 Conclusion

In this paper, we introduce a new four-parameter Lomax distribution called the New Alpha Power Transformed Power Lomax distribution. The new distribution allows for more flexibility in analyzing real-world data sets. The proposed distribution has several characteristics of the NAPTPLx distribution. The density function of the new model contained variable shapes, such as exponentially decreasing and unimodal behavior. Some statistical properties are derived. The parameters of the NAPTPLx distribution are estimated using the MLE method. The behavior of the derived estimators is assessed using a comprehensive simulation study. In addition, Bayesian estimation using the MCMC approach is also utilized to estimate the model parameters. Two data sets from different fields, bladder cancer, and tax returns, are utilized to show the applicability of the proposed distribution. The proposed distribution was analyzed more efficiently as compared to MOPLx, MOLx, PLx, and Lx distributions.

## CRediT authorship contribution statement

**Mahreen Abdullah:** Methodology. **Muhammad Ahsan-ul-Haq:** Writing – original draft, Investigation, Conceptualization. **Abdullah M. Alomair:** Writing – review & editing, Software. **Mohammed A. Alomair:** Visualization, Supervision.

## Declaration of Competing interest

The authors have no conflict of interest.

## Appendix

The pdfs of completive distributions are.

• **Lx:**$f\left(x\right)=\theta \lambda^{\;\theta }{\left(\lambda +x\right)}^{-\theta -1}$
• **PLx:**$f\left(x\right)=\theta \beta \lambda^{\theta }x^{\beta -1}{\left(\lambda +x^{\beta }\right)}^{-\theta -1}$
• **MOLx:**$f\left(x\right)=\frac{\alpha \theta \lambda^{\;\theta }{\left(\lambda +x\right)}^{-\theta -1}}{{\left[1-\left(1-\alpha \right)\lambda^{\theta }{\left(\lambda +x\right)}^{-\theta }\right]}^{2}}$
• **MOPLx:**$f\left(x\right)=\frac{\alpha \theta \beta \lambda^{\theta }x^{\beta -1}{\left(\lambda +x^{\beta }\right)}^{-\theta -1}}{{\left[1-\left(1-\alpha \right)\lambda^{\theta }{\left(\lambda +x^{\beta }\right)}^{-\theta }\right]}^{2}}$
